# Supplementary material for: Eosinophil count trajectories are associated with the prognosis of acute myocardial infarction patients: Insights from ICU data analysis
Source: PLoS One. 2026 Jun 4;21(6):e0349827. doi: 10.1371/journal.pone.0349827 (PMC13235902; doi:10.1371/journal.pone.0349827)
Supplement: S1 Table — (DOCX) [file pone.0349827.s001.docx]

**Table S1. Multicollinearity Diagnostics Using Generalized Variance Inflation Factors (GVIF).**

| **Variables** | **GVIF** |
| --- | --- |
| **Age** | 1.402 |
| **Gender** | 1.090 |
| **BMI** | 1.104 |
| **SBP** | 1.555 |
| **DBP** | 1.629 |
| **HR** | 1.144 |
| **HB** | 1.293 |
| **WBC** | 1.094 |
| **PLT** | 1.078 |
| **Scr** | 1.779 |
| **Bun** | 1.991 |
| **cTnT** | 1.135 |
| **Hypertension** | 1.163 |
| **HF** | 1.168 |
| **AF** | 1.181 |
| **CKD** | 1.423 |
| **DM** | 1.137 |
| **APSIII** | 1.395 |
| **ACEI/ARB** | 1.318 |
| **Beta** | 1.304 |
| **Antiplatelet** | 1.486 |
| **Statin** | 1.449 |
| **PCI** | 1.113 |
| **CABG** | 1.251 |

Abbreviations as in Table 1.
