# Supplementary material for: Eosinophil count trajectories are associated with the prognosis of acute myocardial infarction patients: Insights from ICU data analysis
Source: PLoS One. 2026 Jun 4;21(6):e0349827. doi: 10.1371/journal.pone.0349827 (PMC13235902; doi:10.1371/journal.pone.0349827)
Supplement: S2 Table — (DOCX) [file pone.0349827.s002.docx]

**Table S2. Univariate Cox regression analysis for 28-day and 1-year mortality.**

| **Variables** | **28-day mortality** | | **1-year mortality** | | | |
| --- | --- | --- | --- | --- | --- | --- |
|  | **HR (95%CI)** | **P value** | **HR (95%CI)** | | **P value** | |
| **Age** | 1.02 (1.01 ~ 1.03) | <0.001 | 1.03 (1.02 ~ 1.04) | <0.001 | |  |
| **Gender** |  |  |  |  | |  |
| **Male** | 1.00 (Reference) |  | 1.00 (Reference) |  | |  |
| **Female** | 1.08 (0.88 ~ 1.33) | 0.448 | 1.12 (0.95 ~ 1.31) | 0.183 | |  |
| **BMI** | 1.00 (0.98 ~ 1.02) | 0.850 | 0.98 (0.97 ~ 0.99) | 0.007 | |  |
| **SBP** | 0.99 (0.99 ~ 0.99) | 0.023 | 0.99 (0.99 ~ 0.99) | 0.004 | |  |
| **DBP** | 1.00 (0.99 ~ 1.00) | 0.327 | 1.00 (0.99 ~ 1.00) | 0.082 | |  |
| **HR** | 1.01 (1.01 ~ 1.01) | 0.046 | 1.01 (1.01 ~ 1.01) | 0.015 | |  |
| **HB** | 0.92 (0.88 ~ 0.95) | <0.001 | 0.90 (0.88 ~ 0.93) | <0.001 | |  |
| **WBC** | 1.01 (1.01 ~ 1.01) | 0.014 | 1.01 (1.01 ~ 1.01) | 0.048 | |  |
| **PLT** | 0.99 (0.99 ~ 0.99) | 0.006 | 0.99 (0.99 ~ 0.99) | 0.003 | |  |
| **Scr** | 1.04 (1.01 ~ 1.07) | 0.014 | 1.05 (1.03 ~ 1.07) | <0.001 | |  |
| **Bun** | 1.01 (1.01 ~ 1.01) | <0.001 | 1.01 (1.01 ~ 1.01) | <0.001 | |  |
| **cTnT** | 1.02 (0.99 ~ 1.04) | 0.294 | 1.00 (0.98 ~ 1.03) | 0.961 | |  |
| **Hypertension** |  |  |  |  | |  |
| **No** | 1.00 (Reference) |  | 1.00 (Reference) |  | |  |
| **Yes** | 0.80 (0.62 ~ 1.05) | 0.104 | 0.92 (0.74 ~ 1.14) | 0.437 | |  |
| **HF** |  |  |  |  | |  |
| **No** | 1.00 (Reference) |  | 1.00 (Reference) |  | |  |
| **Yes** | 1.08 (0.88 ~ 1.32) | 0.486 | 1.22 (0.97 ~ 1.54) | 0.086 | |  |
| **AF** |  |  |  |  | |  |
| **No** | 1.00 (Reference) |  | 1.00 (Reference) |  | |  |
| **Yes** | 1.27 (1.03 ~ 1.55) | 0.022 | 1.34 (1.14 ~ 1.56) | <0.001 | |  |
| **CKD** |  |  |  |  | |  |
| **No** | 1.00 (Reference) |  | 1.00 (Reference) |  | |  |
| **Yes** | 1.32 (1.08 ~ 1.63) | 0.008 | 1.57 (1.34 ~ 1.84) | <0.001 | |  |
| **DM** |  |  |  |  | |  |
| **No** | 1.00 (Reference) |  | 1.00 (Reference) |  | |  |
| **Yes** | 0.89 (0.72 ~ 1.09) | 0.241 | 1.03 (0.88 ~ 1.20) | 0.754 | |  |
| **APSIII** | 1.03 (1.02 ~ 1.03) | <0.001 | 1.03 (1.02 ~ 1.03) | <0.001 | |  |
| **ACEI/ARB** |  |  |  |  | |  |
| **No** | 1.00 (Reference) |  | 1.00 (Reference) |  | |  |
| **Yes** | 0.16 (0.12 ~ 0.22) | <0.001 | 0.37 (0.31 ~ 0.44) | <0.001 | |  |
| **Beta** |  |  |  |  | |  |
| **No** | 1.00 (Reference) |  | 1.00 (Reference) |  | |  |
| **Yes** | 0.21 (0.17 ~ 0.26) | <0.001 | 0.30 (0.26 ~ 0.36) | <0.001 | |  |
| **Antiplatelet** |  |  |  |  | |  |
| **No** | 1.00 (Reference) |  | 1.00 (Reference) |  | |  |
| **Yes** | 0.49 (0.39 ~ 0.62) | <0.001 | 0.60 (0.50 ~ 0.73) | <0.001 | |  |
| **Statin** |  |  |  |  | |  |
| **No** | 1.00 (Reference) |  | 1.00 (Reference) |  | |  |
| **Yes** | 0.36 (0.29 ~ 0.45) | <0.001 | 0.48 (0.40 ~ 0.57) | <0.001 | |  |
| **PCI** |  |  |  |  | |  |
| **No** | 1.00 (Reference) |  | 1.00 (Reference) |  | |  |
| **Yes** | 0.58 (0.37 ~ 0.91) | 0.017 | 0.82 (0.61 ~ 1.10) | 0.187 | |  |
| **CABG** |  |  |  |  | |  |
| **No** | 1.00 (Reference) |  | 1.00 (Reference) |  | |  |
| **Yes** | 0.21 (0.13 ~ 0.34) | <0.001 | 0.22 (0.16 ~ 0.31) | <0.001 | |  |

Abbreviations as in Table 1.
