# Supplementary material for: Eosinophil count trajectories are associated with the prognosis of acute myocardial infarction patients: Insights from ICU data analysis
Source: PLoS One. 2026 Jun 4;21(6):e0349827. doi: 10.1371/journal.pone.0349827 (PMC13235902; doi:10.1371/journal.pone.0349827)
Supplement: S3 Table — AIC: Akaike Information Criterion; BIC: Bayesian Information Criteria. (DOCX) [file pone.0349827.s003.docx]

**Table S3. The Group-based Trajectory Modelling (GBTM) parameters (AIC, BIC and class sizes) for EOS count trajectory grouping.**

| **Number of classes** | **AIC** | **BIC** | **Class 1 (%)** | **Class 2 (%)** | **Class 3 (%)** | **Class 4 (%)** | **Class 5 (%)** |  |
| --- | --- | --- | --- | --- | --- | --- | --- | --- |
| **1** | 1000.034 | 1018.958 | 100% | - | - | - | - |  |
| **2** | -4483.106 | -4438.950 | 15.74% | 84.26% | - | - | - |  |
| **3** | -7705.768 | -7642.688 | 35.83% | 13.40% | 50.77% | - | - |  |
| **4** | -8851.013 | -8762.701 | 26.39% | 5.36% | 42.93% | 25.32% | - |  |
| **5** | -9085.617 | -8978.382 | 24.11% | 1.88% | 37.58% | 11.25% | 25.18% |  |

**AIC: Akaike Information Criterion; BIC: Bayesian Information Criteria.**
