# Supplementary material for: Eosinophil count trajectories are associated with the prognosis of acute myocardial infarction patients: Insights from ICU data analysis
Source: PLoS One. 2026 Jun 4;21(6):e0349827. doi: 10.1371/journal.pone.0349827 (PMC13235902; doi:10.1371/journal.pone.0349827)
Supplement: S4 Table — AvePP: Average Posterior Probabilities. (DOCX) [file pone.0349827.s004.docx]

**Table S4. The Group-based Trajectory Modelling (GBTM) parameters (AvePP) for EOS count trajectory grouping.**

| **Number of classes** | **Traj1** | **Traj2** | **Traj3** | **Traj4** | **Traj5** |  |
| --- | --- | --- | --- | --- | --- | --- |
| **1** | 1.0000000 | - | - | - | - |  |
| **2** | 0.9923073 | 0.9983746 | - | - | - |  |
| **3** | 0.9988445 | 0.9959385 | 0.9960837 | - | - |  |
| **4** | 0.9938468 | 0.9895640 | 0.9968160 | 0.9994929 | - |  |
| **5** | 0.9837985 | 0.9951111 | 0.9938883 | 0.9867222 | 0.999694 |  |

**AvePP: Average Posterior Probabilities.**
