# Supplementary material for: Eosinophil count trajectories are associated with the prognosis of acute myocardial infarction patients: Insights from ICU data analysis
Source: PLoS One. 2026 Jun 4;21(6):e0349827. doi: 10.1371/journal.pone.0349827 (PMC13235902; doi:10.1371/journal.pone.0349827)
Supplement: S5 Table — OCC: Odds of Correct Classification. (DOCX) [file pone.0349827.s005.docx]

**Table S5. The Group-based Trajectory Modelling (GBTM) parameters (OCC) for EOS count trajectory grouping.**

| **Number of classes** | **Traj1** | **Traj2** | **Traj3** | **Traj4** | **Traj5** |  |
| --- | --- | --- | --- | --- | --- | --- |
| **1** | - | - | - | - | - |  |
| **2** | 510.3194 | 155.2611 | - | - | - |  |
| **3** | 1543.9491 | 1579.3859 | 247.6455 | - | - |  |
| **4** | 449.8874 | 1680.4365 | 416.1354 | 5816.9834 | - |  |
| **5** | 191.7571 | 10596.8348 | 270.3652 | 583.4193 | 9693.198 |  |

**OCC: Odds of Correct Classification.**
