# Supplementary material for: Eosinophil count trajectories are associated with the prognosis of acute myocardial infarction patients: Insights from ICU data analysis
Source: PLoS One. 2026 Jun 4;21(6):e0349827. doi: 10.1371/journal.pone.0349827 (PMC13235902; doi:10.1371/journal.pone.0349827)
Supplement: S6 Table — Model1: unadjusted; Model2: adjusted for age, gender, BMI; Model3: adjusted for age, gender, BMI, SBP, HR, HB, WBC, PLT, Scr, Bun, cTnT, HF, AF, CKD, APSIII, ACEI/ARB, Beta blocker, Antiplatelet drugs, Statin, PCI, CABG. (DOCX) [file pone.0349827.s006.docx]

**Table S6. The associations of EOS count trajectories with ICU mortality and severe AKI incidence in AMI patients**

|  | **Model1** | | **Model2** | | **Model3** | |
| --- | --- | --- | --- | --- | --- | --- |
|  | **OR (95%CI)** | **P value** | **OR (95%CI)** | **P value** | **OR (95%CI)** | **P value** |
| **ICU mortality** |  |  |  |  |  |  |
| Trajectory1 | Ref |  | Ref |  | Ref |  |
| Trajectory2 | **0.25 (0.14, 0.44)** | **<0.001** | **0.23 (0.13, 0.41)** | **<0.001** | **0.40 (0.20, 0.80)** | **0.010** |
| Trajectory3 | **0.41 (0.31, 0.55)** | **<0.001** | **0.39 (0.29, 0.53)** | **<0.001** | **0.62 (0.42, 0.90)** | **0.012** |
| **Severe AKI incidence** |  |  |  |  |  |  |
| Trajectory1 | Ref |  | Ref |  | Ref |  |
| Trajectory2 | **0.62 (0.44, 0.89)** | **0.009** | **0.58 (0.40, 0.83)** | **0.003** | **0.62 (0.42, 0.92)** | **0.018** |
| Trajectory3 | **0.59 (0.46, 0.75)** | **<0.001** | **0.56 (0.43, 0.72)** | **<0.001** | **0.63 (0.48, 0.82)** | **<0.001** |

Model1: unadjusted.

Model2: adjusted for age, gender, BMI.

Model3: adjusted for age, gender, BMI, SBP, HR, HB, WBC, PLT, Scr, Bun, cTnT, HF, AF, CKD, APSIII, ACEI/ARB, Beta blocker, Antiplatelet drugs, Statin, PCI, CABG.

Abbreviations as in Table 1.
