# Supplementary material for: Eosinophil count trajectories are associated with the prognosis of acute myocardial infarction patients: Insights from ICU data analysis
Source: PLoS One. 2026 Jun 4;21(6):e0349827. doi: 10.1371/journal.pone.0349827 (PMC13235902; doi:10.1371/journal.pone.0349827)
Supplement: S7 Table — Model1: unadjusted; Model2: adjusted for age, gender, BMI; Model3: adjusted for age, gender, BMI, SBP, HR, HB, WBC, PLT, Scr, Bun, cTnT, HF, AF, CKD, APSIII, ACEI/ARB, Beta blocker, Antiplatelet drugs, Statin, PCI, CABG. (DOCX) [file pone.0349827.s007.docx]

**Table S7. Mediation effect of severe AKI on the association between the EOS count trajectories and 28-day mortality**

| **28-day mortality** | **Direct effect** | |  | **Indirect effect** | | | **Proportion mediated (%)** |
| --- | --- | --- | --- | --- | --- | --- | --- |
|  | **Coefficients (95%CI)** | **P value** |  | **Coefficients (95%CI)** | **P value** |  |  |
| **Model 1** | 172.41 (109.69, 257.60) | <0.001 |  | 21.36 (7.70, 41.62) | <0.001 |  | 10.61 (4.39, 18.42) |
| **Model 2** | 174.11 (105.33, 269.68) | <0.001 |  | 22.83 (8.23, 43.52) | <0.001 |  | 11.33 (4.89, 19.45) |
| **Model 3** | 254.85 (108.40, 470.21) | <0.001 |  | 36.46 (2.93, 91.16) | 0.032 |  | 11.87 (1.39, 28.02) |

Model1: unadjusted.

Model2: adjusted for age, gender, BMI.

Model3: adjusted for age, gender, BMI, SBP, HR, HB, WBC, PLT, Scr, Bun, cTnT, HF, AF, CKD, APSIII, ACEI/ARB, Beta blocker, Antiplatelet drugs, Statin, PCI, CABG.

Abbreviations as in Table 1**.**
