# Supplementary material for: Eosinophil count trajectories are associated with the prognosis of acute myocardial infarction patients: Insights from ICU data analysis
Source: PLoS One. 2026 Jun 4;21(6):e0349827. doi: 10.1371/journal.pone.0349827 (PMC13235902; doi:10.1371/journal.pone.0349827)
Supplement: S8 Table — Model1: unadjusted; Model2: adjusted for age, gender, BMI; Model3: adjusted for age, gender, BMI, SBP, HR, HB, WBC, PLT, Scr, Bun, cTnT, HF, AF, CKD, APSIII, ACEI/ARB, Beta blocker, Antiplatelet drugs, Statin, PCI, CABG. (DOCX) [file pone.0349827.s008.docx]

**Table S8. The associations of EOS% trajectories with 28-day and 1-year mortality in AMI patients**

|  | **Model1** | | **Model2** | | **Model3** | |
| --- | --- | --- | --- | --- | --- | --- |
|  | **HR (95%CI)** | **P value** | **HR (95%CI)** | **P value** | **HR (95%CI)** | **P value** |
| **28-day mortality** |  |  |  |  |  |  |
| Trajectory1 | Ref |  | Ref |  | Ref |  |
| Trajectory2 | **0.43 (0.34, 0.54)** | **<0.001** | **0.44 (0.34, 0.55)** | **<0.001** | **0.58 (0.45, 0.74)** | **<0.001** |
| Trajectory3 | **0.33 (0.21, 0.52)** | **<0.001** | **0.32 (0.20, 0.52)** | **<0.001** | **0.49 (0.30, 0.78)** | **0.003** |
| **1-year mortality** |  |  |  |  |  |  |
| Trajectory1 | Ref |  | Ref |  | Ref |  |
| Trajectory2 | **0.55 (0.46, 0.65)** | **<0.001** | **0.56 (0.47, 0.67)** | **<0.001** | **0.72 (0.60, 0.86)** | **<0.001** |
| Trajectory3 | **0.52 (0.38, 0.70)** | **<0.001** | **0.51 (0.37, 0.69)** | **<0.001** | **0.66 (0.48, 0.91)** | **0.010** |

Model1: unadjusted.

Model2: adjusted for age, gender, BMI.

Model3: adjusted for age, gender, BMI, SBP, Heart rate, HB, WBC, PLT, Scr, Bun, cTnT, HF, AF, CKD, APSIII, ACEI/ARB, Beta blocker, Antiplatelet drugs, Statin, PCI, CABG.

Abbreviations as in Table 1.
