# Supplementary figures and images for: Eosinophil count trajectories are associated with the prognosis of acute myocardial infarction patients: Insights from ICU data analysis
Source: PLoS One. 2026 Jun 4;21(6):e0349827. doi: 10.1371/journal.pone.0349827 (PMC13235902; doi:10.1371/journal.pone.0349827)

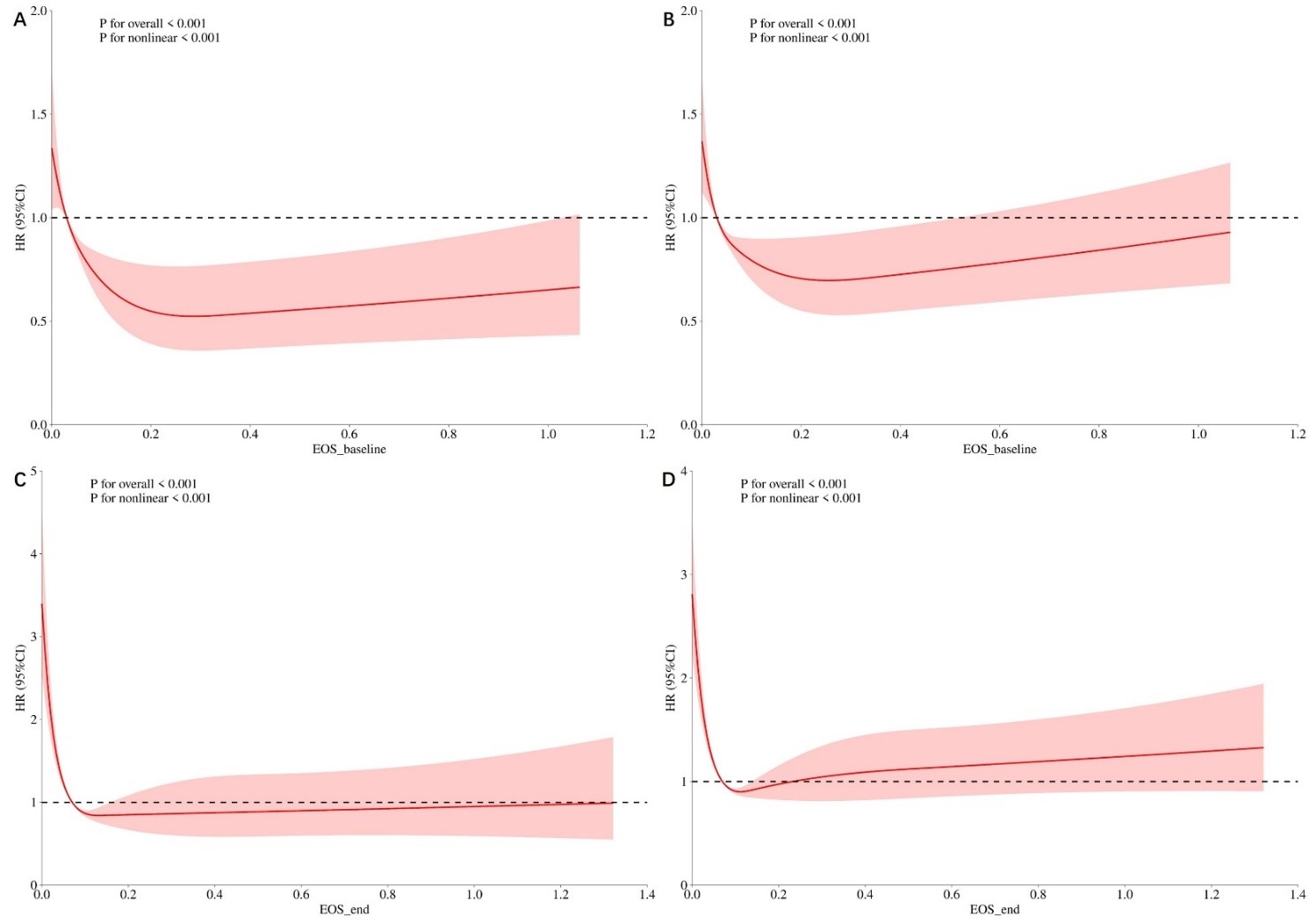

Supplement: S1 Fig — (A) Baseline EOS count and 28-day mortality, (B) Baseline EOS count and 1-year mortality, (C) Last EOS count and 28-day mortality and (D) Last EOS count and 1-year mortality. (JPG) [file pone.0349827.s009.jpg]

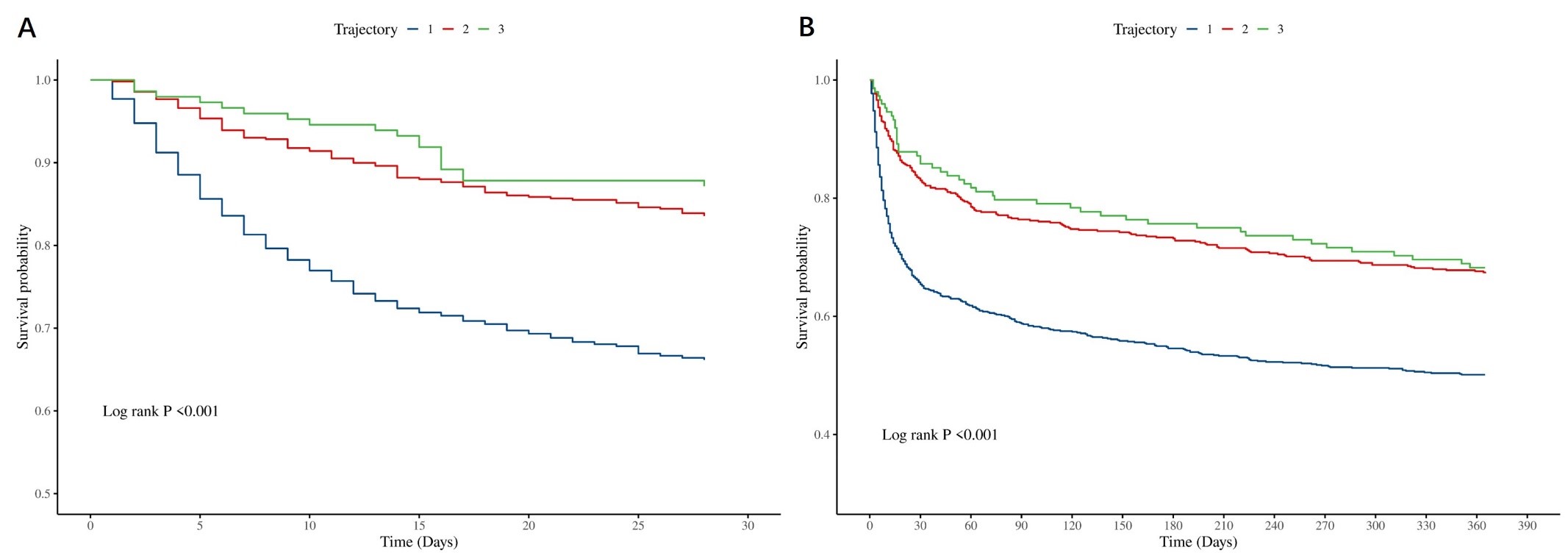

Supplement: S2 Fig — (A) 28-day mortality, (B) 1-year mortality. (JPG) [file pone.0349827.s010.jpg]
