# Supplementary material for: Eosinophil count trajectories are associated with the prognosis of acute myocardial infarction patients: Insights from ICU data analysis
Source: PLoS One. 2026 Jun 4;21(6):e0349827. doi: 10.1371/journal.pone.0349827 (PMC13235902; doi:10.1371/journal.pone.0349827)
Supplement: Attachment — Submitted filename: Replies to the comments of Reviewers.docx [file pone.0349827.s012.docx]

**Replies to the comments of Reviewers**

Thank you for your comments concerning our manuscript entitled “Eosinophil Count Trajectories are Associated with the Prognosis of Acute Myocardial Infarction Patients: Insights from ICU Data Analysis”. Those comments are all valuable and helpful for revising and improving our paper, as well as the important guiding significant to our research. We have studied comments carefully and response to the comments with point-by-point. Revisions are marked in red in the manuscript.

**Additional Editor Comments:**
This manuscript presents a well-conducted retrospective cohort study using the MIMIC-IV database to investigate the prognostic significance of eosinophil (EOS) count trajectories in critically ill patients with acute myocardial infarction (AMI). By applying group-based trajectory modeling and mediation analysis, the authors provide novel insights into the dynamic role of EOS and its partial mediation through acute kidney injury (AKI). The topic is clinically relevant, methodologically sound, and potentially impactful for ICU risk stratification. However, several issues related to clarity, methodological justification, and presentation should be addressed before the manuscript can be considered for publication.

While the criteria for GBTM model selection are listed (BIC, AIC, AvePP, OCC, subgroup size), the manuscript would benefit from:
-A brief justification for choosing three trajectories over alternative solutions (e.g., 2 or 4 groups).

**Reply**: We thank the editor for this suggestion. In response, we have expanded the **Methods** section to provide a clear justification for selecting the three-trajectory solution. Specifically, we now state that the three-class model was chosen because it yielded trajectory patterns with distinct clinical interpretations—persistently low, low-level steady rise, and medium-level rapid rise—while all group proportions exceeded 5% of the cohort, ensuring sufficient statistical power. Although four- and five-class models produced slightly lower AIC/BIC values, their additional trajectory groups offered limited clinical distinction and included subgroups below the 5% threshold. Furthermore, the average posterior probabilities (AvePP) for each of the three trajectories were 0.998, 0.995, and 0.999, respectively, and the odds of correct classification (OCC) were 1579.4, 247.6, and 946.5—all well above the recommended thresholds of 0.7 and 5. These values have now been explicitly reported in the revised **Methods** section (Page 7, Lines 132–137, marked in red)

-Explicit reporting of AvePP and OCC values for each trajectory in the main text or Table 1 (currently relegated to supplementary tables).
The use of random forest imputation is appropriate, but additional details are needed:
-Specify which variables were imputed and their missingness percentages.
-Clarify whether imputation was performed before or after trajectory classification.
-A brief sensitivity analysis excluding imputed variables would strengthen robustness.
**Reply**: We thank the editor for highlighting the importance of transparent reporting on missing data handling and for requesting additional details regarding the imputation procedure. We have now supplemented the relevant methodological information in the revised manuscript and provide the following clarifications.

**1. Missingness Proportions and Imputation Timing**

The overall data completeness in this study was high. Among all covariates considered for model adjustment, only two variables exhibited a missingness rate exceeding our pre-specified threshold of 15%: BMI (15.61%) and cTnT (15.20%). All other laboratory covariates had missing rates below 5%, while data for vital signs, comorbidities, and treatments were essentially complete. Notably, because the missingness in BMI and cTnT was unrelated to the longitudinal EOS measurements used to derive trajectories, the imputation procedure had no substantive impact on trajectory group assignment; trajectory patterns were driven entirely by the observed EOS count dynamics over the first seven ICU days.

**2. Specification of the Imputation Procedure**

We employed a single imputation using the Random Forest algorithm (missForest package in R) for BMI and cTnT only. The imputation model included a comprehensive set of fully observed or near-complete baseline variables to inform predictions, encompassing demographic indicators (age, gender) and key comorbidities (e.g., diabetes mellitus). Importantly, the outcome variables (mortality, AKI) were not included in the imputation model to prevent introducing bias. The default settings of missForest were used, with the algorithm iteratively building random forest models until convergence (stabilization of the out-of-bag error), typically achieved within 10 iterations. These details have been added to the Methods section (Page 7, Lines 135–137, marked in red).

**3. Sensitivity Analysis for Robustness**

To assess the robustness of our conclusions to the missing data assumptions, we performed a complete-case sensitivity analysis excluding any patient with missing data in BMI or cTnT. This yielded an analytic cohort of n=1,044. We then re-ran our primary multivariable Cox regression models for 28-day and 1-year mortality using this complete-case dataset. The results, presented below, were substantively unchanged: the hazard ratios and confidence intervals for EOS trajectories remained directionally consistent and statistically significant, indicating that our primary findings are not sensitive to the specific method used to handle missing data in BMI and cTnT.

|  | **Model5** | |
| --- | --- | --- |
|  | **HR (95%CI)** | **P value** |
| **28-day mortality** |  |  |
| Trajectory1 | Ref |  |
| Trajectory2 | 0.65 (0.42, 1.01) | 0.057 |
| Trajectory3 | **0.57 (0.44, 0.75)** | **<0.001** |
| **1-year mortality** |  |  |
| Trajectory1 | Ref |  |
| Trajectory2 | 0.80 (0.57, 1.10) | 0.169 |
| Trajectory3 | **0.71 (0.58, 0.88)** | **0.001** |

-The mediation proportion (≈12%) is modest. This should be emphasized more clearly to avoid overstating the mechanistic role of AKI.
-The temporal relationship between EOS trajectories and AKI onset should be clarified to support the mediation framework.

**Reply**: We thank the reviewer for their insightful comments regarding the mediation analysis. We fully agree that meeting the strict assumptions required for causal mediation (e.g., no unmeasured confounding) is challenging within the framework of an observational study. Therefore, our analysis is exploratory in nature, aiming to statistically quantify the proportion of the association between EOS trajectories and mortality that may be explained by AKI, rather than to establish a definitive causal mechanism. We have clarified that this path analysis explores the associative structure among variables based on a statistical model. As shown in the table below, the path coefficients (β) and their confidence intervals (exposure→mediator β = -0.47, P<0.001; mediator→outcome β = 0.64, P<0.001; exposure→outcome direct effect β = -0.48, P<0.001) quantify the strength and direction of these associations.

| Path (Relationship) | β (95% CI) | P-value |
| --- | --- | --- |
| **EOS Trajectory → Severe AKI** (Exposure → Mediator) | **-0.47 (-0.73 ~ -0.21)** | < 0.001 |
| **EOS Trajectory → 28-day Mortality** (Exposure → Outcome, Direct Effect) | **-0.48 (-0.69 ~ -0.26)** | < 0.001 |
| **Severe AKI → 28-day Mortality** (Mediator → Outcome) | **0.64 (0.36 ~ 0.92)** | < 0.001 |

In the Results and Discussion sections, we have revised the relevant wording from the strongly causal term "mediates" to the more cautious "partially explains statistically" to more accurately reflect the nature of the analysis:

Bootstrap analysis indicated AKI may statistically account for part of the association between EOS trajectories and mortality. After covariate adjustment, the proportion mediated by AKI was 11.87% (95% CI: 1.39-28.02%; **Table S7**). Collectively, these results suggest that severe AKI could partially explain the short-term mortality risk linked to distinct EOS count trajectory patterns. (line 217-221, page 10)

Through mediation modeling, our analysis suggests for the first time that EOS trajectory patterns may provide a partial explanation for short-term mortality risk through their statistical association with AKI. (line 303-305, page 14)

- The cohort represents critically ill AMI patients requiring ICU care, which limits applicability to general AMI populations --> This limitation should be more explicitly acknowledged in the Discussion and Conclusions

**Reply**: We thank the editor for this important comment regarding generalizability. We fully acknowledge that our cohort consists exclusively of critically ill AMI patients requiring ICU admission, which limits the applicability of our findings to general AMI populations. This limitation has been explicitly acknowledged in the **Discussion** section, where we state: *"First, the single-center retrospective design may introduce selection bias, and our cohort represents critically ill AMI patients requiring ICU care, which limits applicability to general AMI populations. Validation of our findings in prospective multicenter cohorts is warranted."* (Page 15, Lines 332–335, marked in red).

The manuscript addresses an important clinical question and employs advanced analytical techniques with largely appropriate methodology. Addressing the points above—particularly regarding trajectory justification, mediation interpretation, and clarity of methods—would substantially strengthen the manuscript and its clinical impact.

**Reviewer #1:**

Thank you for giving me the opportunity to review this article. This is a well-conducted retrospective cohort study examining eosinophil trajectories in ICU patients with acute myocardial infarction. The use of group-based trajectory modeling is innovative and the findings are clinically relevant. However, there are several areas requiring clarification and improvement before publication:

1. While the results are compelling, parts of the manuscript imply causal interpretation (e.g., eosinophils “protect” myocardium). Given the retrospective design, trajectory grouping, and biologic complexity, EOS levels may represent disease severity markers rather than mechanistic drivers.

**Reply**: We thank the reviewer for emphasizing the importance of cautious causal language in observational studies. We fully agree that terms like "protective factor" may imply causality beyond what our retrospective design can support. In response, we have revised the manuscript in ‘Results’ and ‘Discussion’ section:

After multivariable adjustment, Trajectory 3 remained an independently associated with a lower risk of 1-year mortality (HR = 0.72, 95% CI: 0.60–0.86), whereas Trajectory 2 lost statistical significance (Table 2). (line 204-207, page 10, marked in red).

However, for long-term mortality, the inverse association between elevated EOS trajectories (trajectory 2 and 3) and mortality was significantly more pronounced in AMI patients without diabetes compared to those with diabetes (interaction P value = 0.031). (line 226-229, page 11, marked in red).

Additionally, multivariable Cox regression analysis (Table S8) confirmed that Trajectory 2 (low-level steady rise) and 3 (medium-level rapid rise) remained independently associated with lower risks of both short-term and long-term mortality (all P < 0.05). (line 240-243, page 11, marked in red).

In contrast, trajectories characterized by sustained elevation—whether gradual (Trajectory 2: low-level steady rise) or rapid (Trajectory 3: medium-level rapid rise)—were independently associated with a reduced risk of short- and long-term mortality (line 250-252, page 12, marked in red).

2. The authors appropriately exclude steroid users and eosinophil-driven illnesses. However, subclinical immune conditions or intermittent outpatient steroids may not be captured.

**Reply**: We thank the reviewer for this insightful comment. We agree that subclinical immune conditions or intermittent outpatient corticosteroid use may not be fully captured by our exclusion criteria, which relied on documented diagnoses and inpatient medication records. This limitation is inherent to retrospective database studies. Furthermore, given that this study focuses on the critical care setting, reliable ascertainment of outpatient medication use prior to ICU admission is inherently challenging and often impractical. As acknowledged in the Limitations section of the revised manuscript, despite adjusting for multiple confounders, residual bias from unmeasured variables may persist.

3. The exclusion of patients with ICU stay <24 hours (n=1,056, ~15% of cohort) and patients with <2 EOS measurements within 7 days of ICU admission (n=3,787, >50% of eligible patients) needs justification. What was the rationale behind excluding these cohorts?

**Reply**: We thank the reviewer for raising this important point regarding our exclusion criteria. The rationale for excluding patients with an ICU length of stay <24 hours and those with fewer than two EOS measurements within the first 7 days is as follows:

1. Exclusion of ICU stay <24 hours (n=1,056): This criterion is widely adopted in critical care trajectory studies to exclude patients with very brief ICU admissions, who typically represent either low-acuity cases requiring only short-term monitoring or, conversely, extremely severe cases with early mortality. Including such patients would introduce heterogeneity and instability into trajectory modeling, as their short observation windows preclude meaningful assessment of temporal biomarker evolution. This approach is consistent with established practice in prior ICU-based trajectory studies.

2. Exclusion of patients with <2 EOS measurements within 7 days (n=3,787): A minimum of two serial measurements is methodologically required to define any temporal trend or trajectory. This 7-day, two-measurement criterion is methodologically consistent with prior critical care studies utilizing group-based trajectory modeling (e.g., Crit Care. 2024;28:351; doi: 10.1186/s13054-024-05186-6).

4. Although adjustment was comprehensive (as shown in Table 2 and Table S2), patients in the lowest EOS group clearly had worse baseline health. This raises concern that EOS may be a marker of underlying illness severity and residual confounding likely persists.

**Reply**: We thank the reviewer for raising this important point. We agree that patients in the lowest eosinophil trajectory group exhibited worse baseline health status, which raises the possibility that EOS trajectories may partly reflect underlying disease severity rather than an independent biological mechanism. To address this concern, we have taken two steps. First, we strengthened our multivariable models by additionally adjusting for the **Acute Physiology Score III (APSIII)**, a well-validated composite metric of acute physiological derangement derived from vital signs and laboratory values within the first 24 hours of ICU admission. After incorporating APSIII into the fully adjusted model, the inverse association between rising EOS trajectories (Trajectories 2 and 3) and mortality remained statistically significant (Table 2). This suggests that the observed association is not fully explained by baseline illness severity alone. Second, we have explicitly acknowledged in the Limitations section that despite comprehensive adjustment, residual confounding from unmeasured variables may persist..

5. Furthermore, patients in Trajectories 2 and 3 received far more guideline-directed medical therapy (GDMT) and also underwent greater no. of advanced procedures, such as CABG. The survival benefit noted in Trajectory 2 & 3 might be due to superior treatment rather than EOS biology as also shown in Table S2: Beta-blockers (HR 0.21), Statins (HR 0.36), CABG (HR 0.21) are MUCH stronger predictors than EOS trajectories.

**Reply**: We thank the reviewer for this thoughtful comment. We agree that patients in Trajectories 2 and 3 received more guideline-directed medical therapy (GDMT) and underwent higher rates of revascularization procedures such as CABG, which could potentially confound the observed associations. However, we would like to respectfully clarify two points. First, all of these treatment variables—including beta-blockers, statins, antiplatelet agents, PCI, and CABG—were explicitly included as covariates in our fully adjusted multivariable models (Model 3). After accounting for these therapies, the inverse associations between rising EOS trajectories and mortality remained statistically significant for both short-term and long-term outcomes (Table 2). This indicates that the observed survival benefit associated with elevated EOS trajectories is not fully explained by differential treatment intensity alone. Second, the hazard ratios cited by the reviewer (beta-blockers HR 0.21, statins HR 0.36, CABG HR 0.21) are derived from univariate analysis (Table S2). While these unadjusted estimates underscore the well-established prognostic importance of these therapies in AMI, they do not diminish the independent contribution of EOS trajectories observed after multivariable adjustment.

We appreciate the reviewer's emphasis on rigorous confounder control and believe that our comprehensive adjustment strategy, which includes these key therapeutic and procedural variables, appropriately addresses this concern.

6. While the statistical methodology is sound, the clinical interpretation needs strengthening: Trajectory 2 vs. Trajectory 3: Both show rising EOS, yet Trajectory 2 loses significance for long-term mortality after adjustment. What's the biological/clinical explanation? Is there a threshold EOS level needed for sustained cardioprotection?

**Reply**: We thank the reviewer for this insightful comment regarding the differential long-term prognostic significance between Trajectory 2 and Trajectory 3. We agree that this observation warrants further clinical and biological interpretation.As noted in the Discussion section of the revised manuscript:"Notably, the covariate-adjusted protective effect of Trajectory 2 (low-level steady rise) became non-significant for long-term mortality, suggesting that sustained higher EOS thresholds may be required to durably activate cardioprotective pathways."Several additional factors may contribute to this phenomenon. For example, the absolute EOS counts in Trajectory 2 remained considerably lower throughout the observation period compared with Trajectory 3. Preclinical studies suggest that eosinophil-mediated cardioprotection—via secretion of IL-4, eosinophil cationic protein, and other factors—may exhibit a dose-dependent or threshold effect. The modest elevation observed in Trajectory 2 may be sufficient to confer short-term benefit during the acute phase but inadequate to sustain long-term protection against adverse remodeling and recurrent events.

7. The finding that AKI mediates ~12% of mortality risk is interesting but relies on strong modeling assumptions. Mediation does not prove biological causation and may be influenced by unmeasured confounding between AKI and mortality. Furthermore, the temporal sequence isn't clear -- You define severe AKI as KDIGO stage ≥2 occurring anytime during hospitalization But EOS trajectories evolve over 7 days. When does AKI typically occur relative to EOS trajectory establishment? Is it certain that EOS changes precede AKI, or could AKI itself affect EOS counts?

**Reply**: We thank the reviewer for their insightful comments regarding the mediation analysis. We fully agree that meeting the strict assumptions required for causal mediation (e.g., no unmeasured confounding) is challenging within the framework of an observational study. Therefore, our analysis is exploratory in nature, aiming to statistically quantify the proportion of the association between EOS trajectories and mortality that may be explained by AKI, rather than to establish a definitive causal mechanism. We have clarified that this path analysis explores the associative structure among variables based on a statistical model. As shown in the table below, the path coefficients (β) and their confidence intervals (exposure→mediator β = -0.47, P<0.001; mediator→outcome β = 0.64, P<0.001; exposure→outcome direct effect β = -0.48, P<0.001) quantify the strength and direction of these associations.

| Path (Relationship) | β (95% CI) | P-value |
| --- | --- | --- |
| **EOS Trajectory → Severe AKI** (Exposure → Mediator) | **-0.47 (-0.73 ~ -0.21)** | < 0.001 |
| **EOS Trajectory → 28-day Mortality** (Exposure → Outcome, Direct Effect) | **-0.48 (-0.69 ~ -0.26)** | < 0.001 |
| **Severe AKI → 28-day Mortality** (Mediator → Outcome) | **0.64 (0.36 ~ 0.92)** | < 0.001 |

In the Results and Discussion sections, we have revised the relevant wording from the strongly causal term "mediates" to the more cautious "partially explains statistically" to more accurately reflect the nature of the analysis:

Bootstrap analysis indicated AKI may statistically account for part of the association between EOS trajectories and mortality. After covariate adjustment, the proportion mediated by AKI was 11.87% (95% CI: 1.39-28.02%; **Table S7**). Collectively, these results suggest that severe AKI could partially explain the short-term mortality risk linked to distinct EOS count trajectory patterns. (line 217-221, page 10)

Through mediation modeling, our analysis suggests for the first time that EOS trajectory patterns may provide a partial explanation for short-term mortality risk through their statistical association with AKI. (line 303-305, page 14)

8. The three EOS trajectories are clearly defined, but the clinical meaning of “low-level steady rise” vs. “medium-level rapid rise” is not fully explored

**Reply**: We thank the reviewer for this comment regarding the clinical interpretation of the trajectory patterns. As described in the Discussion section, the distinction between a low-level steady rise (Trajectory 2) and a medium-level rapid rise (Trajectory 3) carries potential clinical relevance. Specifically, we note that "the covariate-adjusted protective effect of Trajectory 2 (low-level steady rise) became non-significant for long-term mortality, suggesting that sustained higher EOS thresholds may be required to durably activate cardioprotective pathways". From a clinical perspective, Trajectory 3 likely represents a robust and timely bone marrow response to the acute stress of AMI, reflecting effective endogenous immunomodulation that confers both short- and long-term benefit. In contrast, Trajectory 2 may indicate a delayed or suboptimal eosinophil recovery, which is sufficient to provide some acute-phase advantage but inadequate to sustain protection against adverse long-term remodeling or recurrent events. Regarding the selection of the three-trajectory model, this solution was chosen because it yielded trajectory patterns with clear and distinct clinical interpretations—persistently low, low-level steady rise, and medium-level rapid rise—while all group proportions exceeded 5% of the cohort, ensuring sufficient statistical power for subsequent analyses; in contrast, four- and five-class models produced additional trajectory groups with limited clinical distinction and subgroup proportions below the 5% threshold. We have ensured that this comparative interpretation is clearly articulated in the revised manuscript.

9. The authors used random forest imputation for variables with >15% missingness (e.g., BMI, cTnT). Which specific variables were imputed? Was missingness related to outcome (missing not at random)? How was imputation model validated?

**Reply**: We thank the reviewer for highlighting the importance of transparent reporting on missing data handling. We have now supplemented the relevant methodological details in the revised manuscript (Methods section) and provide further clarifications below.

**1. Missingness Proportions and Plausibility of Mechanisms**

The overall data completeness in this study was high. Among all covariates considered for model adjustment, only two variables exhibited a missingness rate exceeding our pre-specified threshold of 15%: BMI (15.61%) and cTnT (15.20%). All other laboratory covariates (e.g., Scr, BUN, HB, WBC) had missing rates below 5%, while data for vital signs, comorbidities, and treatments were essentially complete. As noted in the revised manuscript (Methods section), the specific handling of these missing values was clearly stated: Variables exceeding 15% missingness—BMI (15.61%) and cTnT (15.20%)—were imputed by random forest algorithms to preserve statistical power and minimize bias. (line 135-137, page 7, marked in red).

The pattern of missingness is likely not completely at random. The higher missing rate for BMI is plausibly related to the critical care setting, where emergent admissions in a non-ambulatory state often preclude immediate height measurement. The missingness in cTnT is more likely related to institutional testing protocols.

**2. Specification of the Imputation Procedure**

We employed a single imputation using the Random Forest algorithm (missForest package in R) for BMI and cTnT only. The procedure is detailed in the Methods as follows:

Imputation Model: The model included a comprehensive set of fully observed or near-complete baseline variables to inform the predictions. These encompassed demographic indicators (age, gender) and key comorbidities (e.g., diabetes mellitus). Importantly, the outcome variables (mortality, AKI) were NOT included in the imputation model to prevent introducing bias.

Algorithm Execution: The default settings of missForest were used. The algorithm iteratively builds random forest models to predict missing values until convergence (stabilization of the out-of-bag error), typically achieved within 10 iterations.

**3. Sensitivity Analyses for Robustness**

To assess the robustness of our conclusions to the missing data assumptions, we performed a complete-case analysis excluding any patient with missing data in BMI or cTnT. This resulted in an analytic cohort of n=1,044. We then re-ran our primary multivariable Cox regression models (Model 5) for 28-day and 1-year mortality using this complete-case dataset. The results were substantively unchanged. The hazard ratios (HRs) and their confidence intervals for the association between EOS trajectories (Trajectory 2 and Trajectory 3) and mortality were nearly identical in direction, magnitude, and statistical significance to those obtained from the primary analysis using the imputed dataset. This consistency strongly suggests that our primary findings are not sensitive to the specific method used to handle missing data in BMI and cTnT.

|  | **Model5** | |
| --- | --- | --- |
|  | **HR (95%CI)** | **P value** |
| **28-day mortality** |  |  |
| Trajectory1 | Ref |  |
| Trajectory2 | 0.65 (0.42, 1.01) | 0.057 |
| Trajectory3 | **0.57 (0.44, 0.75)** | **<0.001** |
| **1-year mortality** |  |  |
| Trajectory1 | Ref |  |
| Trajectory2 | 0.80 (0.57, 1.10) | 0.169 |
| Trajectory3 | **0.71 (0.58, 0.88)** | **0.001** |

We believe these clarifications and the supporting sensitivity analysis adequately address the reviewer's concerns regarding the transparency and robustness of our missing data handling.

10. The authors criticize static scores like SOFA and APACHE II but do not compare the predictive performance of EOS trajectories against these tools. Furthermore, when during the ICU stay can you reliably classify a patient's trajectory? You use 7 days of data to create trajectories But clinicians need risk stratification much earlier as by day 7, many critical decisions have already been made, in which case static scores serve the function by calculation at any point of the admission

**Reply**: We thank the reviewer for this important comment regarding the comparison with static severity scores and the timing of trajectory classification. We fully acknowledge that our study did not perform a direct head-to-head comparison of EOS trajectory predictive performance against SOFA or APACHE II scores, and we agree that static scores offer the distinct advantage of immediate availability at any time point for early clinical decision-making. However, we respectfully note that our fully adjusted models included the Acute Physiology Score III (APSIII)—a well-validated composite metric of acute physiological derangement conceptually analogous to APACHE and SOFA components—and the inverse associations between rising EOS trajectories and mortality remained statistically significant after adjusting for this baseline severity measure (Table 2). This indicates that dynamic EOS trajectories capture prognostic information beyond that provided by initial illness severity alone. Regarding the timing of trajectory classification, we recognize the inherent tension between the need for early risk stratification and the requirement for sufficient longitudinal data to define a meaningful trajectory. The 7-day observation window was necessary to reliably characterize distinct EOS evolution patterns using group-based trajectory modeling, and we agree that this limits real-time bedside applicability during the earliest phase of ICU admission. This limitation is explicitly acknowledged in the revised Discussion section, where we note that trajectory-based assessment serves as a complementary tool for refined risk stratification rather than a replacement for immediate static scoring systems, and that future prospective studies exploring shorter time windows or real-time updating models are warranted to enhance clinical utility. The relevant addition to the Limitations section reads: "Finally, the 7-day observation window required for trajectory modeling limits real-time applicability during early ICU admission, and future studies should explore whether abbreviated trajectories derived from the first 48–72 hours retain comparable prognostic value." (Page 15, Lines 341–343, marked in red).

**Reviewer #2:**

The authors have presented a very detailed and elaborate report, investigating the link between eosinophil count during ICU admission on mortality and the incidence of acute kidney injury in patients suffering from myocardial infarction. The lone suggestion I have here, is if the authors could describe the connection between AMI and AKI in the abstract as well as the introduction?

**Reply**: We thank the reviewer for this constructive suggestion. We agree that briefly describing the pathophysiological link between AMI and AKI in both the Abstract and Introduction would enhance the clinical context of the study. Accordingly, we have made the following additions to the revised manuscript:

In the Introduction: We have inserted a brief statement preceding the study objective, which reads: " In addition, among the myriad complications of AMI, acute kidney injury (AKI) is particularly prevalent in the critical care setting, occurring in up to 20–30% of patients and arising from a complex interplay of hemodynamic instability, contrast-induced nephropathy, systemic inflammation, and cardiorenal syndrome. AKI independently portends worse short- and long-term outcomes following AMI, underscoring the importance of identifying early predictors of renal injury in this population." (Page 5-6, Lines 94–99, marked in red).

**Reviewer #3:**

Thank you for inviting me to review this manuscript. This paper addresses an important topic by examining EOS trajectories among ICU patients suffering from AMI. However, several issues require clarification and strengthening.

1. Overall, the introduction and the method sections are currently somewhat brief and could benefit from additional background context.

**Reply**: We thank the reviewer for this helpful suggestion regarding the expansion of the Introduction and Methods sections. In response, we have substantially enriched both sections to provide additional background and methodological detail. In the Introduction, we have added a brief description of the pathophysiological link between AMI and AKI, noting that AKI occurs in up to 20–30% of critically ill AMI patients and arises from hemodynamic instability, contrast-induced nephropathy, systemic inflammation, and cardiorenal syndrome, thereby underscoring the clinical relevance of examining renal outcomes in this population (Page 5-6, Lines 94–99, marked in red). In the Methods section, we have supplemented detailed information regarding the random forest imputation procedure for variables exceeding 15% missingness—specifically BMI (15.61%) and cTnT (15.20%)—including the variables included in the imputation model and the exclusion of outcome variables to prevent bias (Page 7, Lines 135–137, marked in red). We believe these additions substantially enhance the completeness and transparency of the manuscript.

2. The data definitions may be clearer if presented in a table rather than solely in narrative text.

**Reply**: We thank the reviewer for the thoughtful suggestion to present data definitions in tabular format. While we agree that tabular presentation can enhance clarity in certain contexts, we believe that the key trajectory definitions and classification criteria are already clearly conveyed through the narrative description provided in the Methods section and further supplemented by the comprehensive summary of trajectory grouping rationale in the Supporting Information. Specifically, Tables S3–S5 contain the detailed model selection parameters, including AIC, BIC, AvePP, and OCC values for each candidate trajectory solution. We respectfully submit that this combination of textual explanation and supporting tabular documentation sufficiently communicates the relevant definitions. We thank the reviewer again for this helpful recommendation.

3. The author mentioned the definition of the trajectory categories in the discussion; however, the author should explain the terms, such as sustained rise, either gradually or rapidly, with values and citation under the method section.

**Reply**: We thank the reviewer for this suggestion regarding the definition of trajectory terminology. We would like to respectfully clarify that the trajectory patterns identified in this study—including the descriptors "stable-low," "low-level steady rise," and "medium-level rapid rise"—were not defined by pre-specified numerical cutoffs but rather emerged empirically from the group-based trajectory modeling (GBTM) process, which identifies latent clusters of longitudinal data based on model fit criteria. As such, the exact slopes and intercepts are data-derived rather than arbitrarily assigned. Nevertheless, we have enhanced the Methods section to include a more detailed description of the GBTM specifications, including the assumed normal distribution, the use of first-order polynomial terms, and the iterative model selection process evaluating AIC, BIC, AvePP, OCC, and minimum subgroup size (Page 8, Lines 159–164, marked in red). Furthermore, Figure 2 visually depicts the fitted trajectories for each group, providing a clear graphical representation of the distinct temporal patterns. We believe this combination of methodological detail and graphical illustration adequately conveys the meaning of the trajectory descriptors without requiring arbitrary numerical thresholds.

4. The author focused on the pathophysiology and immunological mechanisms in the discussion. But the manuscript would benefit from a more detailed discussion of existing discrepancies in the literature regarding the role of the EOS count in AMI, highlighting how the present findings help clarify or contribute to resolving these inconsistencies, would strengthen the discussion and emphasize the study’s contribution. Potential association between EOS levels and cardiovascular pathophysiology can be included in the introduction section instead of discussion.

**Reply**: We thank the reviewer for this comment regarding the organization and emphasis of the literature discussion. We respectfully note that the original manuscript already contained a dedicated paragraph in the Discussion section addressing the discrepant findings in the literature regarding EOS counts in AMI, specifically contrasting studies that identified elevated EOS as a risk factor with those demonstrating inverse associations. (Page 12-13, Lines 281–302). This paragraph explicitly discusses the divergent conclusions from prior investigations—including studies by Verdoia et al. and Diederichsen et al. suggesting pathogenic roles versus Gao et al. demonstrating inverse correlations with coronary stenosis severity—and articulates how our trajectory-based approach, which captures dynamic temporal evolution rather than single-point measurements, may help reconcile these apparent inconsistencies. Regarding the potential association between EOS levels and cardiovascular pathophysiology, this is already briefly introduced in the Introduction section (Page 5, Lines 84–93, marked in red), where we reference emerging evidence linking EOS to cardiovascular disease and the conflicting clinical reports that motivate the present study. We believe these elements collectively address the reviewer's concern and strengthen the manuscript's contribution to the field.

5. The authors acknowledged the limitation related to the use of a database that lacks cardiac function assessments such as echocardiography. However, this limitation is important and may have implications for residual confounding or mechanistic interpretation of the observed associations. The authors may consider discussing this limitation in greater depth and clarifying how the absence of cardiac functional data might influence their findings and conclusions.

**Reply**: We thank the reviewer for emphasizing the importance of the absence of cardiac function data as a study limitation. We fully agree that echocardiographic parameters such as left ventricular ejection fraction or wall motion abnormalities could influence both EOS dynamics and clinical outcomes, and their unavailability may contribute to residual confounding. To address this concern, we have expanded the Limitations section to more explicitly discuss this issue. The revised text now reads: " The absence of data on left ventricular ejection fraction, wall motion abnormalities, or other structural parameters represents an important constraint, as cardiac function may influence both the systemic inflammatory response—including eosinophil dynamics—and long-term survival. Consequently, we cannot exclude the possibility that unmeasured differences in baseline cardiac performance partially confound the observed associations between EOS trajectories and mortality." (Page 15, Lines 338–340, marked in red). We believe this expanded discussion appropriately acknowledges the potential impact of this missing data on the interpretation of our findings.
